# Triglyceride-glucose index: carotid intima-media thickness and cardiovascular risk in a European population

**DOI:** 10.1186/s12933-025-02574-2

**Published:** 2025-01-13

**Authors:** Chiara Pavanello, Massimiliano Ruscica, Sofia Castiglione, Giuliana Germana Mombelli, Antonia Alberti, Laura Calabresi, Cesare Riccardo Sirtori

**Affiliations:** 1 Centro E. Grossi Paoletti, Dipartimento di Scienze Farmacologiche e Biomolecolari “Rodolfo Paoletti”, Università degli Studi di Milano, Milano, Italy; 2 Dyslipidemia Center, SSD Diagnosi e Cure Territoriali Malattie Cardiache, ASST Grande Ospedale Metropolitano Niguarda, Milan, Italy; 3 Dipartimento di Scienze Farmacologiche e Biomolecolari “Rodolfo Paoletti”, Università degli Studi di Milano, Milan, Italy; 4 Department of Cardio-Thoracic-Vascular Diseases, Foundation IRCCS Ca’ Granda Ospedale Maggiore Policlinico, Milan, Italy

**Keywords:** Triglyceride-glucose index, Carotid artery intima-media thickness, Insulin resistance, Coronary artery disease, Longitudinal study

## Abstract

**Background:**

The triglyceride-glucose (TyG) index is now widely recognized as a marker of insulin resistance and has been linked to the development and prognosis of atherosclerotic cardiovascular diseases (ASCVD) in numerous populations, particularly in the Eastern world. Although there are fewer reports from the Western world, and they are sometimes contradictory, the absence of definitive data on the relationship between a raised TyG index and cardiovascular risk suggested the opportunity of testing this biochemical marker against a well-established vascular marker such as the carotid intima media thickness (c-IMT).

**Methods:**

Primary prevention patients were selected from a cohort of individuals who underwent c-IMT measurement between 1984 and 2018 at the Dyslipidemia Center at the ASST Grande Ospedale Metropolitano Niguarda in Milan, Italy. The TyG index was calculated as the Ln [fasting TG (mg/dL)×fasting glucose (mg/dL)/2]. Carotid ultrasonography was performed using echographic measurements of the far walls of the left and right common, internal carotids, and bifurcations. Patients were followed for up to 20 years with periodic evaluation of biochemical parameters. ASCVD events were monitored through hospital records, where all patients were regularly examined.

**Results:**

The analysis included 3108 individuals with a mean age of 54.9 ± 13.1 years. Participants were generally non-obese, with an average BMI of 24.6 ± 3.5 Kg/m^2^. Among the women, 83.1% were postmenopausal. The mean TyG index was 8.65 ± 0.59. There was a significant association between the TyG index and all c-IMT measurements. Those in the highest TyG index quartiles had significantly higher IMT_mean_ and IMT_max_ compared to those in the lower quartiles. These associations were consistent across all vascular sites examined and remained significant after adjusting for all potential confounders. Kaplan-Meier survival analysis revealed an increased incidence of ASCVD events in the two highest TyG index quartiles.

**Conclusions:**

TyG index is a sensitive marker of risk in a European population with moderate ASCVD risk, as assessed by c-IMT measurements, in a large cohort of Lipid Clinic patients.

**Supplementary Information:**

The online version contains supplementary material available at 10.1186/s12933-025-02574-2.

## Introduction

Evaluation of biochemical markers of atherosclerotic cardiovascular (ASCVD) risk has placed at the forefront low-density lipoprotein (LDL) cholesterol, but more recent interest has been placed on triglycerides (TG), high-density lipoprotein (HDL) cholesterol and lipoprotein(a) levels [1–3]. Aside from these well-established lipid/lipoprotein markers, the interest in surrogate markers, particularly of diabetes-associated risk, e.g., insulin resistance (IR), has been growing and offers a number of evaluations directly assessing insulin levels, such as the homeostatic model assessment for insulin resistance (HOMA-IR) index and others [4]. Since these require determination of fasting insulin, not accessible to many laboratories, the potential of the triglyceride-glucose (TyG) index [5] has gained popularity, particularly in view of the need to use generally available data from any patient [6]. Interestingly, in a comparative study the TyG index outperformed the HOMA-IR index in the direct assessment of IR [7, 8].

In spite of the wide availability and the current enthusiasm in this “novel biomarker in the era of cardiometabolic medicine” [6], the use of the TyG index as a marker of ASCVD risk has been mainly restricted to Asian populations [9, 10]. A small number of contributions have been reported from, e.g., European populations. In an Italian population, Neglia et al., indicated TyG index as a reliable outcome index for coronary patients [11]; very recently the Uric Acid Right for Heart Health (URRAH) Project in over 16,000 Italian high-risk patients identified a TyG index cutpoint associated with a significant rise of coronary mortality [12]. In contrast to these, however, pooled data from two major drug based coronary prevention studies, carried out mainly in European countries, only noted a modest elevation of risk in patients with high TyG index, attenuated by the control of LDL-cholesterol [13].

Determination of the carotid intima-media thickness (c-IMT) as a correlate of ASCVD risk factors has been the object of numerous reports, but correlations with TyG index have been provided in very few studies. Our interest in thickness of the intima-media complex dates back to the first methodological study [14], followed by a number of reports investigating correlations with lipids/lipoproteins [15] and platelet reactivity [16]. IMT has been rated a highly reliable ASCVD risk marker is a large US population [17] and has been used by the Italian Collaborative Group in large drug [18] and epidemiological studies [19].

A very recent Chinese study evaluated the progression of c-IMT versus TyG index trajectories, but without providing baseline c-IMT [20]. A positive correlation between c-IMT and TyG index has been reported in Europe by a Hungarian group, however at the aortic level [21]. There is instead the need to investigate c-IMTs from different angles, in particular IMTs of the longitudinal common carotid and of the bifurcations [22]. Evaluation at the latter sites allows, in fact, to calculate the IMT_max_, well correlated with the chronological age of the patient, to be added to the common c-IMTs in a more reliable calculation of risk [23]. In our experience, incidence of the so-called “plaque”, generally identified as a focal thickness exceeding 1.5 mm [24] provides limited information [25]. Use of this last diagnostic endpoint to assess correlation with the TyG index in an Italian population detected only a modest, questionable association [26] and a similar conclusion came out of a very recent Swedish study in a middle-aged population of both genders [25] where “plaque” had a weak association with CV risk factors. We decided, therefore, to leave out this parameter from the vascular findings in the present study.

Determination of the c-IMT, both as a ASCVD risk marker [17] and as a tool to investigate arterial disease progression/regression and the correlated changes in ASCVD risk [27] thus provides essential information in order to evaluate concomitant variations in the TyG index. However, this vascular diagnostic method should be applied using instruments allowing excellent resolution, in order to identify very small differences [28].

In the present report, 3108 consecutive primary prevention patients of an Italian Lipid Clinic, presenting with a wide range of lipoprotein abnormalities, from high LDL-cholesterol, to hypertriglyceridemia, low HDL-cholesterol and diabetes underwent the determination of c-IMT with a highly sensitive equipment. Availability of the TyG index in all of these patients allowed to determine correlations in a population where this variable has been rarely assessed.

## Materials and methods

### Subjects

**Fig. 1:**
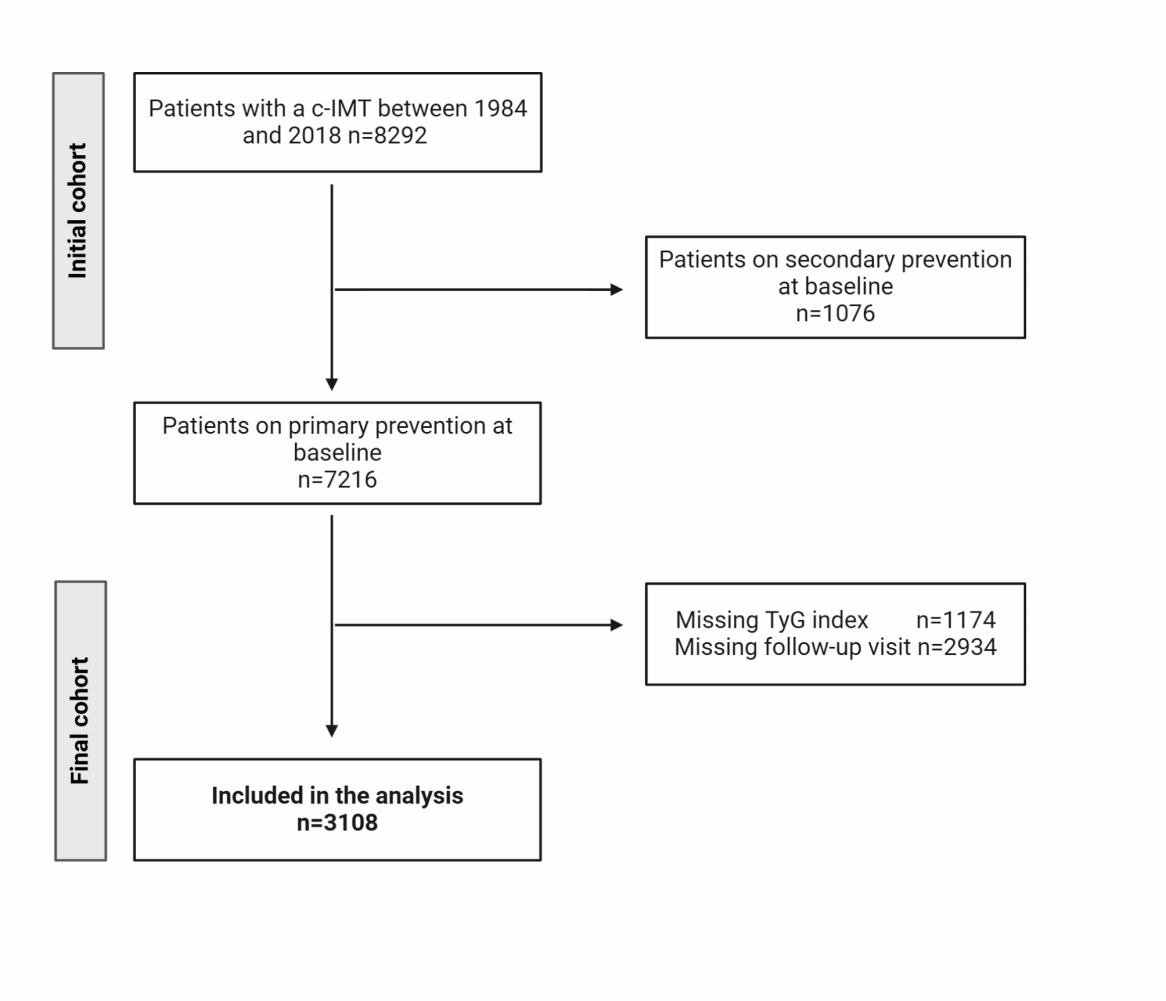
Study flowchart

Participants were retrospectively selected from a cohort of 8292 individuals who underwent a c-IMT measurement between 1984 and 2018 at the Dyslipidemia Center at the Azienda Socio Sanitaria Territoriale (ASST) Grande Ospedale Metropolitano Niguarda in Milan, Italy. Inclusion criteria for the analysis were: (i) primary prevention at the time of the examination or asymptomatic atherosclerosis (ii) quantifiable TyG index (iii) at least one follow-up visit (Fig. 1). The final cohort thus consists of 3108 individuals, 1505 men and 1603 women (Table 1).

**Table 1:** Characteristics of the study population per TyG index quartiles

	Overall	Q1 (6.61–8.24)	Q2 (8.24–8.60)	Q3 (8.60-9.00)	Q4 (9.00-11.62)	*P* value
*n*	3108	777	777	777	777	
Sex (M/W)	1505/1603	254/523	323/454	406/371	522/255	< 0.001
Age (years)	54.9 ± 13.1	51.7 ± 15.3	56.2 ± 12.8	56.3 ± 12.1	55.3 ± 11.3	< 0.001
BMI (Kg/m²)	24.6 ± 3.5	23.0 ± 3.2	24.2 ± 3.2	25.2 ± 3.4	26.2 ± 3.2	< 0.001
Systolic blood pressure (mmHg)	126.7 ± 14.7	122.0 ± 14.3	127.1 ± 14.5	128.9 ± 14.1	128.9 ± 14.8	< 0.001
Diastolic blood pressure (mmHg)	79.0 ± 8.7	76.1 ± 8.5	78.7 ± 8.5	80.0 ± 8.3	81.0 ± 8.5	< 0.001
Post-menopausal women (*n*, %)	1333 (83.1)	381 (72.8)	404 (89.0)	323 (87.1)	225 (88.2)	< 0.001
Active smokers (*n*, %)	618 (19.9)	116 (14.9)	146 (18.8)	156 (20.1)	200 (25.7)	< 0.001
Hypertension (*n*, %)	1389 (44.7)	261 (33.6)	340 (43.8)	364 (46.8)	424 (54.6)	< 0.001
Diabetes (*n*, %)	245 (7.9)	8 (0.1)	30 (3.9)	62 (8.0)	145 (18.7)	< 0.001
Lipid-lowering treatment (*n*, %)	999 (32.1)	244 (31.4)	225 (29.0)	267 (34.4)	263 (33.8)	0.086
Biochemical parameters						
Total cholesterol (mg/dL)	255.8 ± 51.7	252.9 ± 51.7	256.2 ± 48.7	256.8 ± 50.3	257.3 ± 56.0	0.101
Non HDL-cholesterol (mg/dL)	201.2 ± 51.4	187.9 ± 51.9	197.6 ± 47.8	205.3 ± 48.8	214.1 ± 53.3	< 0.001
Triglycerides (mg/dL)	154.9 ± 130.9	72.4 ± 15.3	107.4 ± 16.0	149.6 ± 24.4	290.2 ± 200.2	< 0.001
HDL-cholesterol (mg/dL)	54.6 ± 16.5	65.0 ± 17.8	58.6 ± 14.8	51.6 ± 13.1	43.2 ± 11.2	< 0.001
LDL-cholesterol (mg/dL)	171.7 ± 49.5	173.4 ± 51.5	176.1 ± 47.5	175.4 ± 48.5	161.3 ± 49.0	< 0.001
Fasting blood glucose (mg/dL)	88.6 ± 14.8	81.1 ± 9.1	86.2 ± 10.8	89.5 ± 13.2	97.4 ± 19.1	< 0.001
Uric acid (mg/dL)^#^	5.0 ± 1.3	4.4 ± 1.2	4.7 ± 1.2	5.2 ± 1.3	5.6 ± 1.4	< 0.001
TyG index	8.65 ± 0.59	7.96 ± 0.23	8.42 ± 0.10	8.79 ± 0.11	9.44 ± 0.40	< 0.001

Values are expressed as mean ± standard deviation or number (percentage). Comparisons were performed using the Kruskal-Wallis test for continuous variables and chi-square test for categorical variables. #available for *n =* 3058

BMI, body mass index; HDL, high-density lipoprotein; LDL, low-density lipoprotein

The study was notified to the Ethics Committee of the ASST Grande Ospedale Metropolitano Niguarda. According to the Italian regulation for spontaneous, single-center, observational, retrospective studies, a formal request for an opinion from the Ethics Committee was waived. All participants had provided an oral informed consent. This study was conducted according to the guidelines specified in the Declaration of Helsinki.

### Data collection and definitions

Demographic information (age, sex), clinical history (hypertension, diabetes, menopausal status) and cardiovascular risk factors (smoking habits) were collected at the time of the first visit and retrieved for the present analysis. Hypertension was defined as a systolic blood pressure (SBP) ≥ 140 mmHg and/or diastolic blood pressure (DBP) ≥ 90 mmHg, and/or previous diagnosis of hypertension, and/or use of antihypertensive treatments [29]. Diabetes was defined as fasting blood glucose ≥ 126 mg/dL, previous diagnosis of diabetes, and/or use of hypoglycaemic drugs. In women, mostly menopausal, the thyroid status was verified by thyroid-stimulating hormone (TSH) determination and ultrasound/clinical observation of the presence of thyroid nodules, these being a ground for exclusion.

### Assessment of metabolic parameters

Anthropometric parameters and blood pressure were measured by standard methods. Blood was collected after an overnight fast. Plasma lipids (total cholesterol, HDL-cholesterol, TG), blood glucose and uric acid were determined with certified enzymatic methods by using a Roche diagnostics Cobas c311 autoanalyzer. Plasma LDL-cholesterol was calculated using the Friedewald formula. The TyG index was calculated as the Ln [fasting TG (mg/dL)×fasting glucose (mg/dL)/2]. All assessments were performed within 1 month before or after the ultrasonographic measurements.

### Ultrasonographic variables

Carotid ultrasonography was conducted as previously described [30]. In the course of the study, methodology was changed in order to take advantage of a more modern piece of equipment, allowing more rapid acquisition of echographic data. Up to 2017, echography was carried out with the Technos, (Esaote, Genoa, Italy) equipped with a 5- to 10-MHz linear array probe, calibrated with a phantom at baseline and checked after 1 year. The far walls of the left and right common carotids, bifurcations, and internal carotid arteries were visualized at 3 scan angles (lateral, anterior, and posterior) and recorded using a dedicated software (M’Ath, Metris SRL, Argenteuil, France) [31]. From 2017, 18 patients underwent the same procedures using an EPIQ 7 Philips device (Convex Array, Frequency: 2–6 MHz, Max Depth: 40 cm), following careful standardization to ensure optimal compatibility of echographic findings between the two machines. This approach allowed for a pooled evaluation of the data. The repeatability of c-IMT measurements has been documented previously, and a guideline has been provided [32].

### Cardiovascular events

Occurrence of ASCVD events was followed through the Niguarda Hospital database, all patients being regularly monitored at this Institution. Defined ASCVD events were: myocardial infarction, angina pectoris, ischemic stroke, transient ischemic attack, new diagnosis of intermittent claudication, or surgical interventions for revascularization of coronary or peripheral arteries. These endpoints were assessed at 2, 4, 10 years and at the longest follow-up interval (up to 20 years). Carotid procedures were not included among endpoints, as these may be directly related to the qualifying carotid ultrasound investigation at study entry or during follow-up. Angina pectoris and myocardial infarction were diagnosed according to the Fourth Universal Definition of Myocardial Infarction [33]. All events were validated by our local specialists (GM, AA) through medical records and death certificates. In the case of disagreement, copies of documents were sent to an outside designated specialist in our Cardiology Department, unaware of clinical history and c-IMT data, for adjudication.

### Statistical analyses

Analyses were performed in SPSS version 28.0 and GraphPad Prism 10.1. Descriptive statistics are reported as means and standard deviations (SD) for numerical variables, and as number and percentages for categorical variables. Variables were tested for normality before analyses. The population was stratified according to TyG index quartiles. Numerical variables across quartiles were compared using one-way ANOVA or Kruskal-Wallis test, according to their distributions; categorical variables were compared using the Chi-square test. The selected statistical evaluation for each finding is reported in the Figures/Tables. The relationship between TyG index quartiles and c-IMT variables were first tested by ANOVA for trend. Subsequently, a general linear model (GLM) was used to adjust for potential confounders, including age, sex, BMI, hypertension, diabetes, use of lipid-lowering medication, smoking status, non-HDL cholesterol. Polynomial contrast analysis was performed and linear, quadratic, and cubic trends were tested. Kaplan-Meier survival curves were generated to evaluate the cumulative incidence of ASCVD events, stratified by quartiles of the TyG index. Differences between survival curves were compared using the log-rank test. Cox proportional hazards regression models were subsequently applied to estimate the hazard ratios (HRs) and 95% confidence intervals (CIs) for ASCVD events across TyG index quartiles. The lowest quartile was used as the reference category. The time scale used for the Cox models was time from baseline evaluation. Participants who did not experience an ASCVD event during the study period were censored at the time of the last follow-up. All tests were two-sided and *P* values < 0.05 were regarded as significant.

## Results

### Baseline patient characteristics

**Fig. 2:**
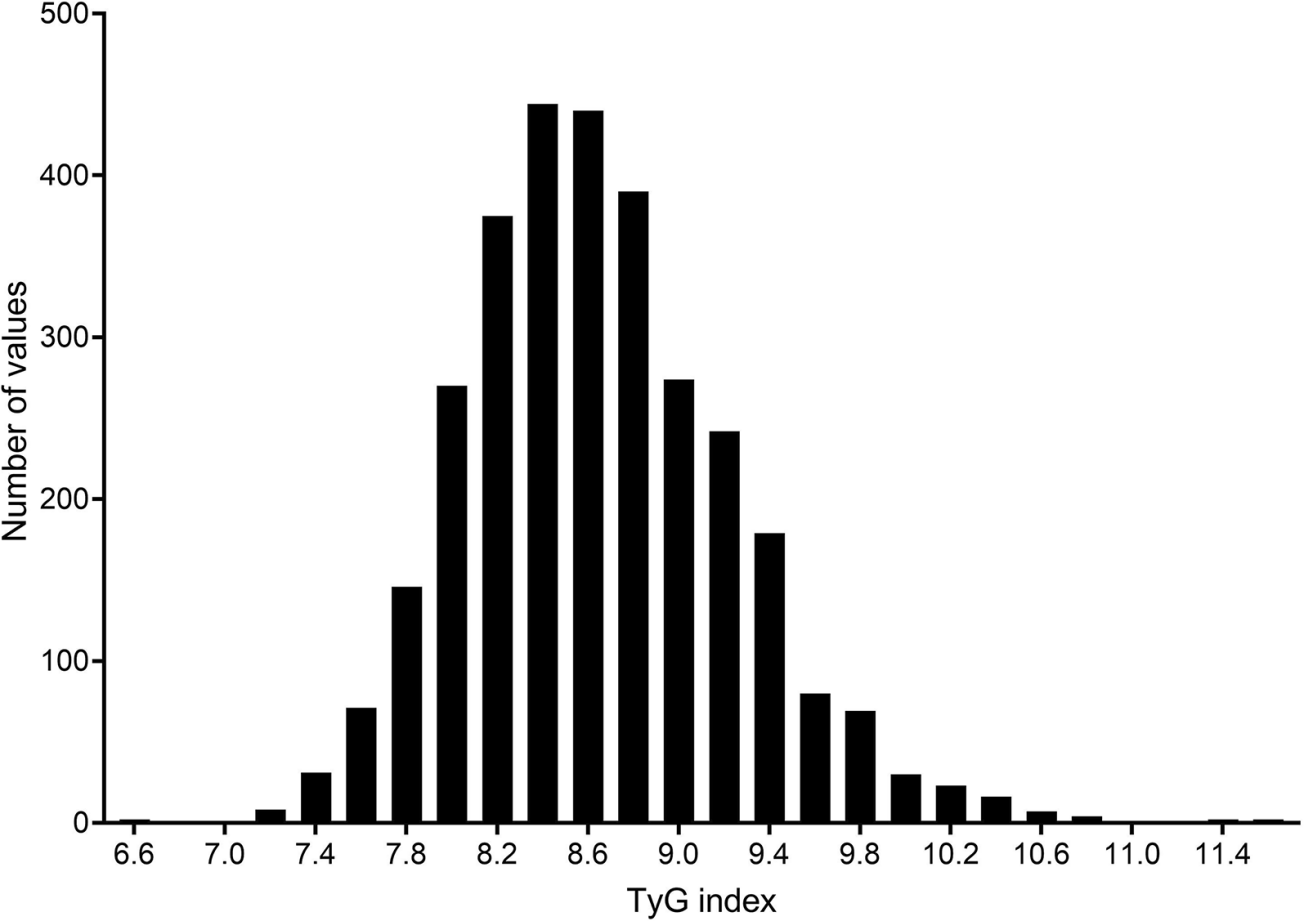
Distribution of TyG index in the study population

A total of 3108 individuals (1505 men and 1603 women) were included in the analysis. The characteristics of the population at baseline are presented in Table 1 and population characteristics stratified by decade of enrolment were summarized in Supplementary Table 1. The mean age was 54.9 ± 13.1 years and patients were generally non-obese (mean BMI 24.6 ± 3.5 Kg/m^2^). Among women, 83.2% were postmenopausal with normal thyroid function. None of the patients were categorized as alcohol abusers. Regarding classical cardiovascular risk factors, one-fifth of the participants were current smokers, 44.7% were hypertensive, and 7.9% were diabetic. At the time of enrollment, just over a quarter of the subjects (32.1%) were already receiving lipid-lowering therapy. The mean TyG index was 8.65 ± 0.59 and TyG index distribution is shown in Fig. 2. The mean TyG index was significantly higher in men compared to women (8.81 ± 0.62 vs. 8.50 ± 0.53, respectively *P* < 0.001). As expected for patients recruited in a Dyslipidemia Center, total cholesterol and LDL-cholesterol were above the normal range with mean values of 255.8 ± 51.7 mg/dL and 171.7 ± 49.5 mg/dL, respectively (reference values < 220 mg/dL and < 116 mg/dL) [34]. Plasma TGs were slightly increased (161.7 ± 156.5 mg/dL, reference value < 150 mg/dL), whereas HDL-cholesterol was within the normal range (54.6 ± 16.5 mg/dL, reference value > 40 mg/dL for men and > 50 mg/dL for women). Only a minority of subjects (*n* = 245) having diabetes, the mean blood glucose level was within the normal range (reference value < 100 mg/dL). Plasma uric acid levels were in the normal range (reference value: 2.6–7.2 mg/dL). TyG index was significantly correlated with all measured laboratory and clinical variables. Interestingly, a strong inverse correlation was observed between the TyG index and HDL-cholesterol (ρ -0.519, *P* < 0.001) (Supplemental Table 2). When analyzing correlations separately for men and women, no significant differences were observed between the two groups. However, it is noteworthy that total cholesterol did not show a significant correlation with the TyG index in men, whereas it did in women (Supplemental Table 2). Patients reporting daily alcohol intake > 2 drinks per day or 14 per week (drink equivalent to 10 mL ethanol) were excluded; regular physical exercise was reported only by a small minority of participants. When stratified according to the quartiles of the TyG index, subjects in the highest TyG index quartile had more pathological metabolic phenotypes, including higher BMI, total cholesterol, lower HDL-cholesterol, higher TG and glycemia. Subjects with a higher TyG index were more hypertensive, diabetics and more frequently smokers.

### TyG index and carotid IMT

**Fig. 3:**
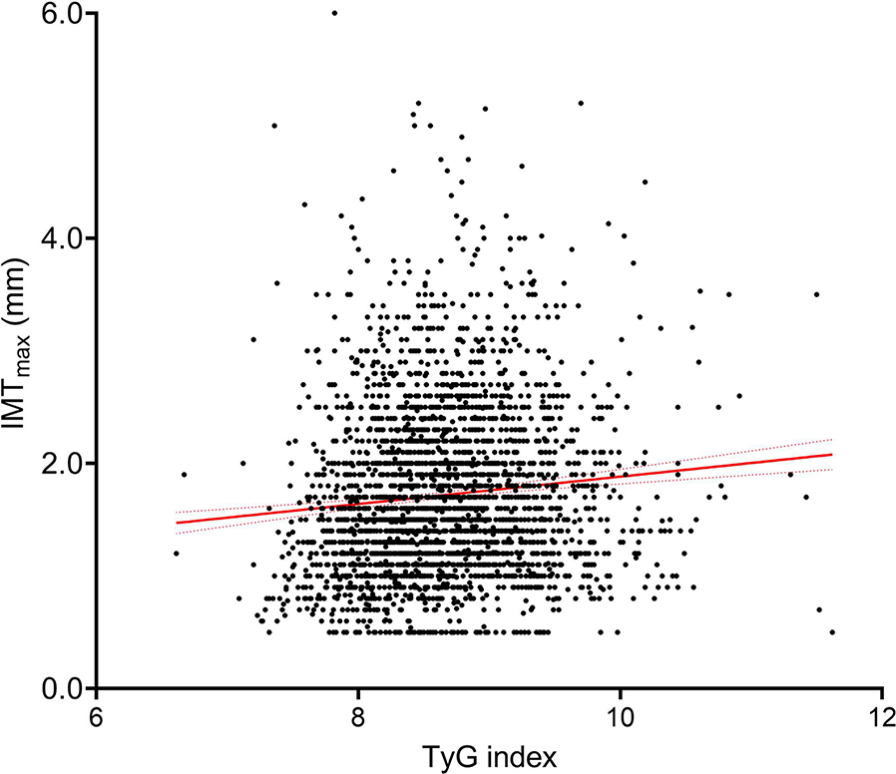
Correlation between TyG index and maximum carotid intima-media thickness n=3108, Spearman's correlation ρ=0.117 P < 0.001

Table 2 presents the ultrasonographic measurements stratified by quartiles of TyG index. In the whole group, the TyG index was significantly associated with all measured variables of c-IMT (Supplemental Table 3, Fig. 3). The correlations between TyG quartiles and ultrasound variables were stronger in women than in men, suggesting that TyG index may have a more consistent association with c-IMT in women (Supplemental Table 3). The wide distribution of data, however in a relatively narrow range for both c-IMT and TyG index leads to a relatively modest slope of the correlation line (Fig. 3). Participants in the higher TyG index quartiles exhibited significantly raised IMT_mean_ and IMT_max_, compared to the lowest quartile. Polynomial contrast analysis revealed a significant linear association between TyG index quartiles and c-IMT, with no significant quadratic or cubic trends. These associations remained consistent across all assessed vascular sites, underscoring the potential role of TyG index as a marker for subclinical atherosclerosis. The associations for mean and mean-max IMTs lost significance after adjustment for age, whereas significances were maintained for BIF-IMT_max_ (*P =* 0.005), IMT_max_ (*P* = 0.009) and IMT_mean−max_ (*P* = 0.011) (Table 2). However, when adjusted for all potential confounders (age, sex, BMI, hypertension, diabetes, lipid-lowering medication, smoking status, non-HDL cholesterol, etc.), TyG index remained significantly positively associated with all IMT variables, except for BIF-IMT_max_ and IMT_max_ (Table 2).

**Table 2:** Carotid IMT per TyG index quartiles

Ultrasonic Variables	Q 1 *n* = 777	Q2 *n* = 777	Q3 *n* = 777	Q4 *n* = 777	*P* trend	Age-adjusted *P* trend	Age and sex- adjusted *P* trend	^#^Fully adjusted *P* trend
CC-IMT_mean_ (mm)	0.886 (0.868–0.904)	0.942 (0.920–0.963)	0.942* (0.920–0.963)	0.935* (0.915–0.954)	< 0.001	0.642	0.524	< 0.001
BIF-IMT_mean_ (mm)	1.104 (1.074–1.134)	1.199 (1.169–1.230)	1.228 (1.195–1.261)	1.214* (1.183–1.245)	< 0.001	0.054	0.874	0.025
ICA-IMT_mean_ (mm)	0.926 (0.900-0.951)	0.970* (0.944–0.997)	0.997* (0.969–1.025)	0.996* (0.968–1.024)	< 0.001	0.173	0.417	0.010
IMT_mean_ (mm)	0.972 (0.950–0.994)	1.037 (1.015–1.059)	1.057* (1.034–1.081)	1.048* (1.025–1.071)	< 0.001	0.121	0.752	< 0.001
CC-IMT_max_ (mm)	1.078 (1.049–1.108)	1.146 (1.117–1.175)	1.183 (1.151–1.214)	1.146* (1.115–1.177)	< 0.001	0.137	0.202	< 0.001
BIF-IMT_max_ (mm)	1.451 (1.404–1.499)	1.614 (1.566–1.663)	1.661 (1.609–1.713)	1.647 (1.597–1.697)	< 0.001	0.005	0.576	0.324
ICA-IMT_max_ (mm)	1.258 (1.210–1.305)	1.329* (1.281–1.377)	1.398 (1.348–1.449)	1.384* (1.335–1.433)	< 0.001	0.068	0.324	0.023
IMT_max_ (mm)	1.577 (1.526–1.629)	1.732 (1.682–1.782)	1.789 (1.735–1.843)	1.776 (1.722–1.829)	< 0.001	0.009	0.743	0.193
IMT_mean−max_ (mm)	1.262 (1.227–1.298)	1.363 (1.227–1.298)	1.414 (1.376–1.425)	1.392* (1.356–1.428)	< 0.001	0.011	0.513	0.008

Results are expressed as means (95% CI). BIF indicates bifurcation; CC, common carotid; CI, confidence interval; ICA, internal carotid artery; IMT, intima-media thickness. *P trend* value represents the unadjusted ANOVA test for trend, assessing the linear association between TyG quartiles and c-IMT variables. The adjusted *P* values are derived from the general linear model, accounting for the effects of covariates ^#^Adjusted for age, sex, BMI, hypertension, diabetes, smoking, lipid-lowering treatment, non HDL-cholesterol. * *P* < 0.05 vs. Q1, assessed by pairwise comparison

### ASCVD risks according to TyG index quartiles

**Fig. 4:**
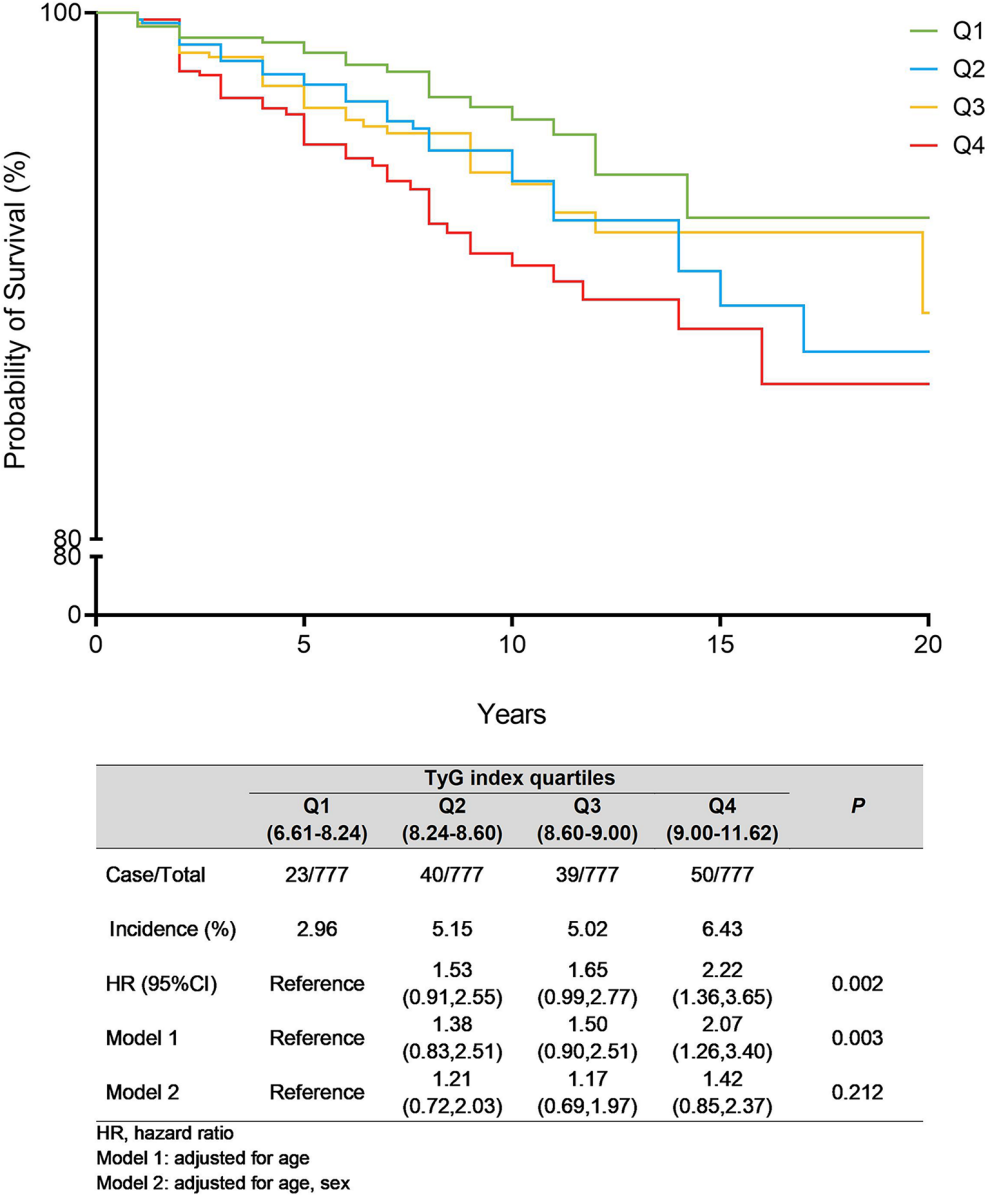
Kaplan-Meier curves for ASCVD event during 20-year follow-up stratified by baseline TyG index quartiles; n=3108 P Cox regression =0.0002

A total of 152 subjects (13.0%) experienced an ASCVD event (median follow-up, 6.59 years [3.42–10.5]). Twenty-three (2.96%) ASCVD events were recorded in the lowest TyG index quartile, 40 (5.15%) in the second and 39 (5.0%) and 50 (6.4%) in the third and fourth, respectively with, however, differences in the occurrence over time (Fig. 4). Coronary heart disease was the most frequent event, ranging from 15 cases in Q1 to 34 in Q4, while myocardial infarction showed a progressive increase, peaking at 25 cases in Q4. Cerebrovascular events, including ischemic stroke and transient ischemic attacks, exhibited inconsistent patterns, while peripheral arterial disease and related revascularizations were relatively stable across quartiles (Table 3). Cox-regression analysis showed that the incidence of ASCVD events differed among TyG index quartiles (*P* = 0.002), and this difference remained significant after adjustment for age (*P* = 0.005) (Fig. 4).

**Table 3:** Incidence of ASCVD events according to TyG index quartiles

	TyG index quartiles			
	Q1 (6.61–8.24)	Q2 (8.24–8.60)	Q3 (8.60-9.00)	Q4 (9.00-11.62)
Case/Total	23/777	40/777	39/777	50/777
**Coronary Heart Disease**	**15 (65.2)**	**30 (75.0)**	**33 (84.6)**	**34 (68.0)**
Myocardial infarction	5 (21.7)	10 (25.0)	9 (23.1)	25 (50.0)
Angina pectoris	4 (17.4)	5 (12.5)	7 (17.9)	1 (2.0)
Coronary artery revascularizations	6 (26.1)	15 (37.5)	17 (43.6)	8 (16.0)
**Cerebrovascular events**	**7 (30.4)**	**27 (25.0)**	**2 (5.1)**	**15 (30.0)**
Ischemic stroke	0	2 (5.0)	0	6 (12.0)
Transient ischemic attack	5 (21.7)	5 (12.5)	0	7 (14.0)
**Peripheral Arterial Disease**	**3 (13.0)**	**3 (7.5)**	**6 (15.4)**	**3 (6.0)**
Peripheral artery revascularization	3 (13.0)	3 (7.5)	6 (15.4)	3 (6.0)

Bold inidcates the macrocategories of ASCVD

Data are expressed as number of events (%)

## Discussion

Assessment of ASCVD risk in large populations is mainly based on the standard biochemical markers, with the addition of variables such as blood pressure, body weight and smoking. Diagnosis of IR has become of major interest in CV risk calculations [35], being associated with metabolic abnormalities such as obesity or the metabolic syndrome. As an initially potential alternative to the direct determination of IR, the TyG index has slowly grown to become a standard evaluation parameter for the determination of ASCVD risk, particularly in Eastern countries. Studies in the West have provided at times controversial findings. In particular, the very recent report by Haring et al., [13] indicated that a higher TyG index in 29960 patients with known atherosclerotic disease was associated with just a moderately raised ASCVD risk in stable patients; risk being attenuated when LDL-cholesterol levels are controlled.

In view of still unclear position of this risk marker in the European population and, in particular, its possible association with better established risk markers, we found it of interest to test the correlation of the TyG index with the c-IMT. Having been responsible for the initial development of this latter diagnostic technique [14], we and many other investigators [17, 18, 36] constantly found it to be a very sensitive marker of the ASCVD risk. The TyG index/IMT correlation was thus tested in association with a number of ASCVD risk variables in this European population, characterized by a relatively moderate ASCVD risk [37].

Earlier work by Japanese authors had indicated that the TyG index can be clearly influenced by the trajectory of risk factors such as hypertension [38]. In this report we determined the mean and maximal c-IMT with sensitive procedures. Recent evidence indicates that particularly the maximal IMT provides a most sensitive marker of ASCVD risk and that changes in this marker also give an excellent prediction of ASCVD events [27]. We had the opportunity, in addition, of following these individuals over a number of years, thus giving an indication of ASCVD risk over time and its relationship with the c-IMT markers.

In the presented series of Lipid Clinic patients recruited in a European area, characterized by moderate CV risk, there was clear evidence of a direct correlation between c-IMT, investigated at different anatomical locations and as IMT_max_, IMT_mean_ and IMT_mean−max,_ and severity of the TyG index alterations. Distributions of this biochemical risk index did not appear to differ markedly from those observed in other populations, thus confirming its reliability in the assessment of risk in a wider geographical area. Association of risk with higher IMT_max_ was well maintained after correction for age, but lost significance after correction for age.

A sex-associated ASCVD risk for TyG index quartiles has never been the object of addressed investigations, but a very recent US study on TyG index in middle-aged and older populations [39] indicated a somewhat reduced ASCVD risk in females. A higher TyG index-associated risk was however reported recently in post-menopausal women, apparently independent of menopausal age [40]. In the present series the majority of women were post-menopausal.

While the association of raised c-IMT with ASCVD risk has been clearly established, that of raised TyG index in low-risk populations, such as the present one, is still debated. From the analysis of the data, with a follow-up of over 20 years, there appear to be differences among patients in the TyG index quartiles. When comparing the incidence in the lowest vs. the highest quartile, these differed significantly with an almost two-fold higher incidence in the latter. Interestingly, a similar approach was very recently followed by Zhang et al. [41], in a large series of coronary patients without standard modifiable risk factors. In these, the TyG index, in a similar range as in the present study, was divided into tertiles. The highest incidence of cardiac and cerebrovascular events, as well as revascularizations, was found in the highest tertiles, the other two behaving similarly. A similar conclusion was reached by Wu et al. [42], investigating, however, the occurrence of carotid plaques, and again indicating a clear rise of risk in the highest vs the lowest TyG index quartile.

The present study had as a major objective that of evaluating a seldom determined marker in a very large, low-risk European population. Participants mainly had moderate total and LDL-cholesterol elevations, as expectable in a Lipid Clinic series, and BMI was within normal limits for a European population, not different from, e.g., a Chinese study on the TyG index [43]. Although this last finding appears not to be associated, in this series, to a blood pressure elevation, it may not exclude a significant impact of IR, a major predictor of CV events, generally best associated with the TyG index [44]; it rather appears to identify TyG index as an independent risk marker.

The excellent correlation of c-IMT with all major CV risk markers, as the conclusion of a detailed investigation on this risk marker in a low-risk population, indicates it as well suited to identify patients in need for further risk reduction or at low event probability [45]. In the case of the TyG index, information from clinical studies on c-IMT has been limited. Aside from the study on c-IMT progression [20], not providing, however, baseline data, an earlier report [46] had shown excellent correlations between the TyG index an c-IMT both mean and maximum, in patients with ischemic stroke. Data from the US based ARIC Study indicated a 40% risk rise in the development of peripheral arterial disease in the upper quartile of the TyG index [47].

The mechanisms linking the TyG index with raised c-IMT are of course of complex unraveling, but several possible explanations may be proposed. A time-dependent increase in the TyG index may lead to stable oxidative stress due to hypertriglyceridemia, causing endothelial dysfunction and contributing to the initiation and progression of atherosclerosis [48]. In parallel, exposure to hyperglycemia may promote the glycation of platelet proteins, enhancing platelet reactivity in an aspirin resistant condition and potentially raising ASCVD risk [49]. The association of insulin and insulin-like growth factor I levels, generally not monitored in these studies, with a gradual rise in IR, may lead to cellular proliferation and reduced apoptosis, potentially contributing to both atheromatosis and potentially to cancer [50]. Interestingly, by the use of a more sophisticated risk marker, such as the TyG index/HDL-C ratio, an excellent association with coronary artery calcification could be recently assessed [51]. This study is among the first to explore the association between long-term TyG index trajectories and all-cause and CVD mortality in a large national cohort. Similarly, the Korea National Statistical Office, leveraging a robust sample size and linking mortality data with official records from the Korea National Statistical Office, captured dynamic changes in TyG index over time, providing a comprehensive understanding of its impact on mortality risk [52]. The above quoted Italian URRAH Project described a significant correlation between TyG levels, above a well-defined cutpoint and coronary mortality, with an interesting link with uric acid elevations [12].

Among limitations of the present study are the relatively small number of patients from a limited geographical area, although similar studies, object of prior publications, frequently reported numbers of 1000 patients or less [53]. This low-risk primary prevention population obviously resulted in a relatively small number of ASCVD events. The study was retrospective, presenting results from a long-term follow-up, with c-IMT evaluation performed only during the initial assessment. The population was, however, well characterized and all patients had a long follow-up at the same clinical organization. Finally, due to the cross-sectional design, establishing causality between the TyG index and c-IMT was precluded. A possible change of the TyG index over time was not determined although it is our plan to investigate these patients also at future intervals, assessing both c-IMT and TyG index changes.

Other limitations should be noted. The study population was recruited through a Lipid Clinic, which may have introduced a selection bias. This Lipid Clinic, however, is the same place, however, where the IMT technology was originally developed [14]. While alcohol abuse was assessed and not reported for any patient, the lack of specific information on dietary variables may prevent a comprehensive evaluation of their potential impact on TyG index. The selected population comes from Southern Europe, seldom the object of this kind of study, although this was the major interest in carrying out the project. The very early start of the study did not consider variables such as the abdominal or waist circumferences or life habits such as physical exercise, for which no adequate accessible software was available at the time. The potential of the c-IMT in predicting CV events was limited by the small number of such events. The change in IMT technology, related to a minority of the followed population (less than 1%), although potentially a limit, will allow us to carry out a longer follow-up, possibly considering other variables, not studied in the present report. Furthermore, because nonfasting lipid profile measurements are used more and more commonly in clinical practice, it is equally important to confirm the findings of the present study in nonfasting conditions [54].

From the overall evaluation of the present study, it appears that the TyG index is a sensitive marker of ASCVD risk in a European population characterised by a relatively low incidence of ASCVD risk. This conclusion stems from a large series of individuals undergoing a c-IMT determination. From the large examined series of Lipid Clinic patients, there does not appear to be a clear link with IR, in view of the overall normality of BMI and blood pressure, thus supporting the hypothesis that a raised TyG index can be an isolated marker of risk in this as in other populations [55].

## Electronic supplementary material

Below is the link to the electronic supplementary material.

Supplementary Material 1

## Data Availability

Data are available upon reasonable request to the corresponding author.

## Abbreviations

ASCVD: Atherosclerotic cardiovascular disease
LDL: Low-density lipoprotein
TG: Triglycerides
HDL: High-density lipoprotein
IR: Insulin resistance
HOMA-IR: Homeostatic model assessment for insulin resistance
TyG: Triglyceride-glucose
c-IMT: Carotid intima-media thickness
SBP: Systolic blood pressure
DBP: Diastolic blood pressure
BMI: Body mass index
BIF: Bifurcation
CC: Common carotid
ICA: Internal carotid artery
