# Supplementary material for: Triglyceride-glucose index: carotid intima-media thickness and cardiovascular risk in a European population
Source: Cardiovasc Diabetol. 2025 Jan 13;24:17. doi: 10.1186/s12933-025-02574-2 (PMC11731386; doi:10.1186/s12933-025-02574-2)
Supplement: Supplementary file 1 — Supplementary Material 1 [file 12933_2025_2574_MOESM1_ESM.docx]

**TRIGLYCERIDE-GLUCOSE INDEX: CAROTID INTIMA-MEDIA THICKNESS AND CARDIOVASCULAR RISK IN A EUROPEAN POPULATION**

Chiara Pavanello^1,2*^, Massimiliano Ruscica^3,4^, Sofia Castiglione^1,2^, Giuliana Germana Mombelli^2^, Antonia Alberti^2^, Laura Calabresi^1^, Cesare Riccardo Sirtori^1,3*^

**Supplemental Table 1.** Characteristics of the study population stratified by decade of enrolment

|  | 1^st^ decade  (1984-1993) | 2^nd^ decade  (1994-2003) | 3^rd^ decade  (2004-2013) | 4^th^ decade  (2014-2018) | *P* value |
| --- | --- | --- | --- | --- | --- |
| *n* | 196 | 433 | 2155 | 324 |  |
| Sex (M/W) | 103/93 | 229/204 | 1024/1131 | 149/175 | 0.099 |
| Age (years) | 51.8 ± 10.6 | 56.1 ± 12.6 | 55.5 ± 13.0 | 50.9 ± 14.3 | < 0.001 |
| BMI (Kg/m²) | 23.5 ± 2.6 | 24.7 ± 3.4 | 24.7 ± 3.5 | 24.8 ± 3.6 | < 0.001 |
| Systolic blood pressure (mmHg) | 128.0 ± 14.3 | 131.4 ± 15.1 | 126.3 ± 14.7 | 123.0 ± 13.0 | < 0.001 |
| Diastolic blood pressure (mmHg) | 81.3 ± 9.0 | 80.4 ± 8.9 | 78.6 ± 8.6 | 78.2 ± 7.6 | < 0.001 |
| Post-menopausal women (N, %) | 86 (92.5) | 174 (85.3) | 950 (84.0) | 123 (70.3) | < 0.001 |
| Active smokers (N, %) | 36 (18.4) | 99 (22.3) | 416 (19.3) | 67 (20.7) | 0.350 |
| Hypertension (N, %) | 90 (45.9) | 238 (55.0) | 950 (44.1) | 111 (34.4) | < 0.001 |
| Diabetes (N, %) | 8 (4.1) | 48 (11.1) | 175 (8.1) | 14 (4.3) | 0.001 |
| Lipid-lowering treatment (N, %) | 58 (29.6) | 59 (13.6) | 170 (7.9) | 28 (8.6) | <0.001 |
| Biochemical parameters |  |  |  |  |  |
| Total cholesterol (mg/dL) | 274 ± 61.7 | 252.7 ± 44.7 | 254.2 ± 51.5 | 259.7 ± 53.4 | < 0.001 |
| Non HDL-cholesterol (mg/dL) | 222.2 ± 61.8 | 201.4 ± 45.1 | 198.7 ± 50.6 | 204.6 ± 54.0 | < 0.001 |
| Triglycerides (mg/dL) | 140.5 ± 70.3 | 165.2 ± 172.2 | 152.6 ± 127.7 | 165.0 ± 114.4 | 0.054 |
| HDL-cholesterol (mg/dL) | 51.7 ± 13.1 | 51.3 ± 14.7 | 55.4 ± 17.0 | 55.2 ± 16.5 | < 0.001 |
| LDL-cholesterol (mg/dL) | 194.2 ± 62.4 | 170.3 ± 45.4 | 169.8 ± 47.9 | 172.6 ± 52.9 | < 0.001 |
| Fasting blood glucose (mg/dL) | 91.3 ± 10.6 | 94.6 ± 14.1 | 87.5 ± 14.8 | 85.5 ± 15.5 | < 0.001 |
| Uric acid (mg/dL) | 5.0 ± 1.7 | 4.6 ± 1.3 | 5.0 ± 1.3 | 5.1 ± 1.4 | < 0.001 |
| TyG index | 8.7 ± 0.5 | 8.8 ± 0.6 | 8.6 ± 0.6 | 8.7 ± 0.6 | < 0.001 |
| Ultrasonographic variables |  |  |  |  |  |
| CC-IMT_mean_ (mm) | 0.667  (0.632 - 0.701) | 0.847  (0.822 - 0.872) | 0.948  (0.936 - 0.959) | 1.049  (1.021 - 1.077) | < 0.001 |
| BIF-IMT_mean_ (mm) | 0.836  (0.785 - 0.886) | 1.108  (1.064 - 1.151) | 1.223  (1.205 - 1.242) | 1.257  (1.210 - 1.305) | < 0.001 |
| ICA-IMT_mean_ (mm) | 0.636  (0.606 - 0.666) | 0.888  (0.854 - 0.923) | 0.998  (0.983 - 1.014) | 1.115  (1.069 - 1.161) | < 0.001 |
| IMT_mean_ (mm) | 0.713  (0.681 - 0.745) | 0.948  (0.918 - 0.977) | 1.057  (1.043 - 1.070) | 1.140  (1.103 - 1.178) | < 0.001 |
| CC-IMT_max_ (mm) | 0.800  (0.746 - 0.854) | 1.088  (1.045 - 1.132) | 1.167  (1.150 - 1.185) | 1.216  (1.173 - 1.260) | < 0.001 |
| BIF-IMT_max_ (mm) | 1.223  (1.125 - 1.322) | 1.617  (1.538 - 1.697) | 1.622  (1.594 - 1.650) | 1.595  (1.518 - 1.672) | < 0.001 |
| ICA-IMT_max_ (mm) | 0.858  (0.778 - 0.939) | 1.303  (1.233 - 1.373) | 1.373  (1.345 - 1.401) | 1.483  (1.399 - 1.567) | < 0.001 |
| IMT_max_ (mm) | 1.330  (1.231 - 1.429) | 1.767  (1.685 - 1.849) | 1.745  (1.715 - 1.775) | 1.710  (1.627 - 1.794) | < 0.001 |
| IMT_mean-max_ (mm) | 0.961  (0.900 - 1.021) | 1.336  (1.283 - 1.389) | 1.387  (1.367 - 1.408) | 1.432  (1.370 - 1.493) | < 0.001 |

Values are expressed as mean ± standard deviation or number (percentage). Comparisons were performed using the Kruskal-Wallis test for continuous variables and chi-square test for categorical variables. Ultrasonographic variables are expressed as means (95% CI). BIF indicates bifurcation; BMI, body mass index; CC, common carotid; CI, confidence interval; HDL, high-density lipoprotein; ICA, internal carotid artery; IMT, intima-media thickness; LDL, low-density lipoprotein.

**Supplemental Table 2.** Spearman’s correlations between clinical variables and TyG index

| 1. Women *(n=1603)* |  |  |
| --- | --- | --- |
| Clinical Variables | ρ | *P* |
| Age | 0.180 | <0.001 |
| BMI | 0.374 | <0.001 |
| Systolic blood pressure | 0.247 | <0.001 |
| Diastolic blood pressure | 0.227 | <0.001 |
| Total cholesterol | 0.160 | <0.001 |
| HDL-cholesterol | -0.459 | <0.001 |
| LDL-cholesterol | 0.095 | <0.001 |
| non HDL-cholesterol | 0.325 | <0.001 |
| 1. Men (*n*=*1504)* |  |  |
| Clinical Variables | ρ | *P* |
| Age | 0.150 | <0.001 |
| BMI | 0.303 | <0.001 |
| Systolic blood pressure | 0.146 | <0.001 |
| Diastolic blood pressure | 0.166 | <0.001 |
| Total cholesterol | 0.034 | 0.189 |
| HDL-cholesterol | -0.478 | <0.001 |
| LDL-cholesterol | -0.203 | <0.001 |
| non HDL-cholesterol | 0.167 | <0.001 |
| C) All (*n*=3*108)* |  |  |
| Clinical Variables | ρ | *P* |
| Age | 0.065 | <0.001 |
| BMI | 0.380 | <0.001 |
| Systolic blood pressure | 0.178 | <0.001 |
| Diastolic blood pressure | 0.208 | <0.001 |
| Total cholesterol | 0.046 | 0.046 |
| HDL-cholesterol | -0.519 | <0.001 |
| LDL-cholesterol | -0.070 | <0.001 |
| non HDL-cholesterol | 0.238 | <0.001 |

BMI, body mass index. Spearman's rank correlation coefficients (ρ) and corresponding *P* values are reported.

**Supplemental Table 3.** Spearman’s correlations between carotid IMT variables and TyG index

| 1. Women *(n=1603)* |  |  |  |
| --- | --- | --- | --- |
| Ultrasonic Variables | Mean ± SD (mm) | ρ | *P* |
| CC-IMT_mean_ | 0.928 ± 0.260 | 0.129 | <0.001 |
| BIF-IMT_mean_ | 1.194 ± 0.432 | 0.159 | <0.001 |
| ICA-IMT_mean_ | 0.972 ± 0.381 | 0.113 | <0.001 |
| IMT_mean_ | 1.031 ± 0.312 | 0.155 | <0.001 |
| CC-IMT_max_ | 1.129 ± 0.401 | 0.136 | <0.001 |
| BIF-IMT_max_ | 1.129 ± 0.401 | 0.177 | <0.001 |
| ICA-IMT_max_ | 1.341 ± 0.678 | 0.118 | <0.001 |
| IMT_max_ | 1.722 ± 0.697 | 0.177 | <0.001 |
| IMT_mean-max_ | 1.358 ± 0.492 | 0.173 | <0.001 |
| B) Men (*n*=*1504)* |  |  |  |
| Ultrasonic Variables | Mean ± SD (mm) | ρ | *P* |
| CC-IMT_mean_ | 0.925 ± 0.295 | 0.028 | 0.275 |
| BIF-IMT_mean_ | 1.178 ± 0.460 | 0.075 | 0.004 |
| ICA-IMT_mean_ | 0.973 ± 0.386 | 0.047 | 0.068 |
| IMT_mean_ | 1.026 ± 0.337 | 0.059 | 0.023 |
| CC-IMT_max_ | 1.148 ± 0.458 | 0.029 | 0.263 |
| BIF-IMT_max_ | 1.584 ± 0.742 | 0.083 | 0.001 |
| ICA-IMT_max_ | 1.343 ± 0.712 | 0.073 | 0.004 |
| IMT_max_ | 1.715 ± 0.795 | 0.084 | 0.001 |
| IMT_mean-max_ | 1.358 ± 0.540 | 0.073 | 0.005 |
| C) All (*n*=3*108)* |  |  |  |
| Ultrasonic Variables | Mean ± SD (mm) | ρ | *P* |
| CC-IMT_mean_ | 0.926±0.278 | 0.068 | <0.001 |
| BIF-IMT_mean_ | 1.186±0.446 | 0.102 | <0.001 |
| ICA-IMT_mean_ | 0.972±0.384 | 0.077 | <0.001 |
| IMT_mean_ | 1.028±0.324 | 0.094 | <0.001 |
| CC-IMT_max_ | 1.138±0.430 | 0.081 | <0.001 |
| BIF-IMT_max_ | 1.593±0.708 | 0.115 | <0.001 |
| ICA-IMT_max_ | 1.342±0.695 | 0.093 | <0.001 |
| IMT_max_ | 1.719±0.746 | 0.117 | <0.001 |
| IMT_mean-max_ | 1.358±0.515 | 0.112 | <0.001 |

BIF indicates bifurcation; CC, common carotid; CI, confidence interval; ICA, internal carotid artery; IMT, intima-media thickness. Spearman's rank correlation coefficients (ρ) and corresponding *P* values are reported.
